# Noniatrogenic Urethral Trauma in Children: A Narrative Review

**DOI:** 10.1016/j.euros.2025.09.016

**Published:** 2025-10-13

**Authors:** Rianne J.M. Lammers, Tudor Enache, Sebastien Faraj, Mieke Waterschoot, Sibel Tiryaki

**Affiliations:** a Department of Urology, University Medical Center Groningen, Groningen, The Netherlands; b Department of Pediatric Surgery, Hôpital Femme-Mère-Enfant, Lyon, France; c Department of Pediatric Surgery and Urology, Sorbonne Université, Armand Trousseau Hospital, AP-HP. Paris, France; d Department of Urology, Ghent University Hospital, Ghent, Belgium; e Department of Pediatric Surgery, Division of Pediatric Urology, Ege University, Izmir, Turkey

**Keywords:** Child, Treatment outcomes, Urethral stricture, Urethral injury, Urethral surgery

## Abstract

**Background and objective:**

Pediatric urethral trauma is rare, and treatment recommendations are often extrapolated from evidence for adults. We conducted a systematic review of the literature to evaluate management strategies, outcomes, and the quality of reporting for pediatric urethral trauma.

**Methods:**

We followed the Preferred Reporting Items for Systematic Reviews and Meta-Analyses (PRISMA) guidelines and identified 31 relevant studies published since 1990. Studies were eligible for inclusions if they reported results for five or more patients with at least 3 mo of follow-up for noniatrogenic urethral trauma. Methodological quality was assessed using the Methodological Index for Nonrandomized Studies (MINORS) tool.

**Key findings and limitations:**

The quality of evidence was low: most studies were retrospective single-center cohorts and there was inconsistent reporting of management and outcomes. Open reconstruction was reported more frequently than endoscopic management. Across treatment modalities, the overall stricture rate was ∼20%. Continence and sexual function outcomes were generally favorable. However, data on repeat surgery and quality of life were insufficient for firm conclusions.

**Conclusions and clinical implications:**

The most striking finding from our review is the poor quality and inconsistency of the literature on pediatric urethral trauma. As well as summarizing the outcome data available, we provide a table of recommendations for improving future studies, including the use of standardized definitions, validated pediatric patient-reported outcome measures, and consistent long-term follow-up.

**Patient summary:**

We looked at the evidence on treatments and outcomes after accidental damage to the urethra in children. No definitive conclusions can be drawn because the quality of the research is low and inconsistent. We provide advice for future research.

## Take Home Message

Current evidence on pediatric urethral trauma is fragmented, but clear opportunities exist to improve the quality and comparability of future research. We provide structured recommendations to guide that process.

## 1 Introduction

Urethral trauma among children is rare and accounts for just 3% of traumatic genitourinary injuries in this population [1,2].Treatment recommendations are mainly extrapolated from data for adults. The question is whether it is correct to extrapolate evidence for adults to children, as the latter have unique anatomic differences that need to be taken into account when considering treatment, such as more immature bones and a higher, more intra-abdominal bladder position. These lead to specific variations in urethral trauma in comparison to adults [2]. In addition, the trauma effect in children is different to that in adults: children experience more unstable pelvic fractures, leading to urethral displacement and bladder rupture [3]. Prepubertal girls are more prone to urethral trauma with pelvic fractures owing to the lack of estrogen-related protection [3]. Urethral trauma is a major cause of urethral strictures: 34% of pediatric urethral strictures have a traumatic etiology [1]. This situation highlights the need for a distinct approach to the management of urethral trauma in children and an evaluation of pediatric outcomes, which prompted us to conduct a systematic review.

## 2 Methods

We followed the Preferred Reporting Items for Systematic Reviews and Meta-Analyses (PRISMA) and conducted a review of the literature using the EMBASE, PubMed, Web of Science, and Scopus databases. The protocol was registered in the PROSPERO database (CRD42023378468).

The PICO (Population, Comparator, Intervention, Outcome) question was framed as follows:

– Population: children with urethral trauma (boys and girls; anterior and posterior injury).
– Intervention: delayed open reconstruction (gold standard in adults).
– Comparators: early open reconstruction, conservative management, endoscopic management.
– Outcomes:
  o Primary: urinary continence, sexual function, stricture rate.
  o Secondary: need for repeat surgery and quality of life (QoL).

The optimal search strategy was identified by testing the ability to retrieve two key control studies [4,5]. The search string was “pediatric urethral trauma”, with the following limitations applied:

– Child/age from birth up to 18 yr.
– Only literature published from 1990 onwards (because of changes in guidelines and operation techniques).
– Only articles in English, Dutch, German, France, Turkish, Russian, or Romanian (the languages spoken in our group).
– Follow-up of at least 3 mo.
– Exclusion of case series and studies with fewer than five cases.
– Exclusion of hypospadias and iatrogenic causes of urethral trauma.

Full-text reading was carried out by two authors per article. One other author (R.J.M.L.) was the independent controller, and checked for reproduction and reliability. If there was a disagreement, a third independent author made the final decision. After group discussion, a data extraction form was set up. Two authors (M.W., S.F.) also checked unpublished data and ongoing studies. Owing to the limited quality of the literature available, a meta-analysis was not feasible; therefore, we present a narrative review.

### 2.1 Statistical analysis

Data are reported as the median or mean for continuous variable, and the frequency and percentage for categorical variables. Nonparametric tests (Mann-Whitney U test or Fisher’s exact test) were used for data analysis. A *p* value <0.05 was considered statistically significant.

### 2.2 Quality assessment

The Methodological Index for Nonrandomized Studies (MINORS) tool [6] was used to evaluate the quality of the studies. This tool gives a maximum of 16 points for noncomparative and 24 for comparative studies.

## 3 Results

**Fig. 1:**
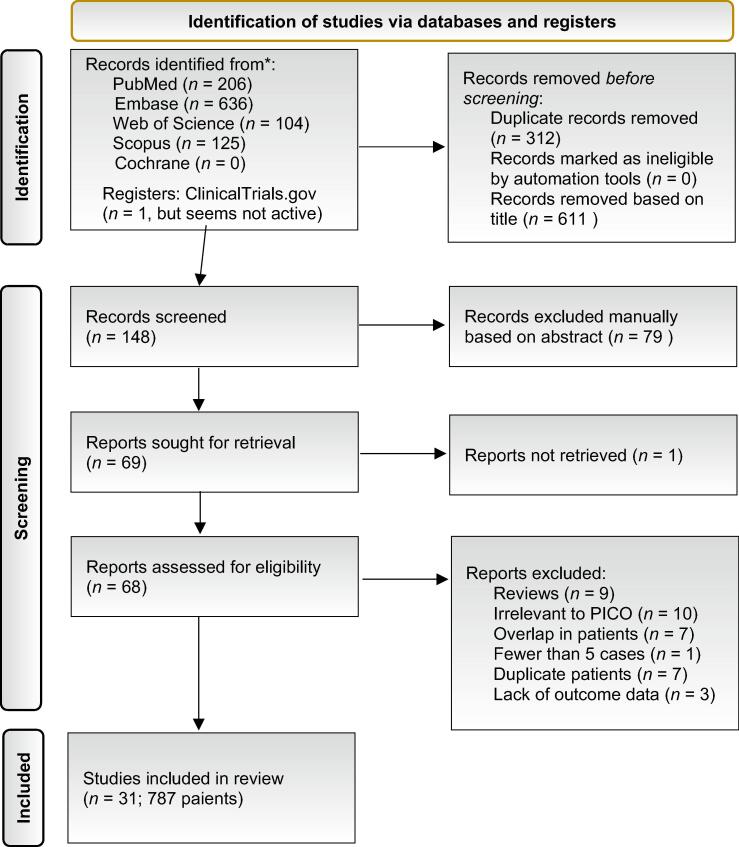
Preferred Reporting Items for Systematic Reviews and Meta-Analyses (PRISMA) flow diagram. PICO = Population, Intervention, Comparator, Outcome.

We identified 1071 articles, of which 68 were considered eligible on the basis of their title and abstract. After applying the inclusion and exclusion criteria, 31 articles involving a total of 787 patients were included in the review [4,5,[7], [8], [9], [10], [11], [12], [13], [14], [15], [16], [17], [18], [19], [20], [21], [22], [23], [24], [25], [26], [27], [28], [29], [30], [31], [32], [33], [34], [35]]. Figure 1 shows the PRISMA flowchart.

### 3.1 Quality assessment

No multicenter studies were identified. Only two articles were nonrandomized comparative studies [11,25]; the rest were cohort studies or case series. All studies but one [25] were retrospective. The mean MINORS score was 8 points (range 4–15; Supplementary Table 1). No relationship was found between the quality of the articles and outcomes.

### 3.2 Early versus late open reconstruction

A total of 30/31 articles addressed open reconstruction. One article exclusively reported on endoscopic realignment [28] according to a protocol that included delayed repair, which was not ultimately required. For the purpose of this review, we defined “early open reconstruction” as any open surgical approach performed immediately after trauma, as there is no clear definition in the literature. Early open reconstruction was the sole method in one study [18], 20 reported only on delayed repair [4,5,[7], [8], [9],[12], [13], [14], [15], [16], [17],^19^,^20^,^24^,^27^,[29], [30], [31], [32],^35^], eight compared early versus delayed open reconstruction [10,11,21,23,25,26,33,34], and one did not specify the timing of surgery [22].

Where data permitted stratification, subgroup analyses were conducted (boys vs girls; anterior vs posterior urethral injury).

#### 3.2.1 Studies including both early and delayed open reconstruction

Of the eight articles evaluating both early and delayed open reconstruction, four included only boys [7,21,23,25], one focused exclusively on girls [26], and three included both sexes [10,11,34]. Among the studies involving boys, six specifically addressed posterior urethral trauma [10,11,21,23,25,33], while one reported on both anterior and posterior urethral trauma [34].

In total, 78 boys underwent early open surgical management, with techniques ranging from simple primary anastomosis to complex reconstruction. Delayed open reconstruction was reported for 56 patients, typically performed 1–6 mo after trauma [10,21,33]. In other studies, the timing was not specified.

With regard to outcomes, an overall repeat surgical procedure was needed in 57 patients (42% of the pooled cohort). These secondary procedures included endoscopic intervention (balloon dilation; urethrotomy) and repeat urethroplasty, with or without buccal mucosa. No further subgroup analysis was conducted.

#### 3.2.2 Studies including only early open reconstruction

Gündoĝdu et al [18] reported results for 11 boys and one girl. In all cases, urethral realignment on a Foley catheter via an open abdominal approach was preferred. No repeat surgery was required.

#### 3.2.3 Studies including only late open reconstruction

The 20 studies reporting on only late open reconstruction involved a total of 611, of whom only 3% were girls. The largest cohort was described by Sreeranga et al [32], with 192 patients (181 boys, 11 girls). Eleven studies reported on posterior urethral trauma [7,8,12,15,16,20,24,27,30,31,35], one addressed anterior urethral trauma [9], and eight reported data for both [4,5,13,14,17,19,29,32]. Table 1 provides an overview of the results for boys. Three studies reported results for only girls or data that allowed separate analysis by sex [9,12,32] (Table 2).

**Table 1:** Overview of studies reporting on late open reconstruction in boys

Author	Year	Country	Trauma location	Patients	Technique and approach used	Repeat procedures
Abdalla [7]	2008	Egypt	Posterior	5	5 posterior parasagittal pararectal perineal approach	None reported
Aggarwal [8]	2011	India	Posterior	34	Primary group: 10 perineal approach with iPBT, 13 abdominoperineal TPA	1 complex Monti reconstruction, 1 sUTP, 1 UDL, 1 cystolithotomy
					Redo group: 1 pure perineal approach, 11 TPA, 7 sUTP	
Basiri [12]	2002	Iran	Posterior	7	7 UTP via abdominal approach with CPD	None reported
Chaudhari [13]	2021	India	Both posterior and anterior	14	14 transperineal UTP	2 UTP
Chukwubuike [14]	2020	Nigeria	Both posterior and anterior	1	11 transperineal UTP	1 UTP
Das [15]	2004	India	Posterior	10	Urethral anastomosis: 4 transperineal approach, 6 transperineal TPA, 4 omental flap	1 UDL
El-Sheikh [16]	2008	Egypt	Posterior	15	Urethral reconstruction via perineal approach only	1 DVIU, 2 UTP via APA
Garg [17]	2019	India	Posterior and anterior	33	PUA: 27 perineal approach, 6 PBT; 4 Mitrofanoff, 1 Badenoch; 1 UTP via TPA + preputial flap	Not specifically mentioned, but 1 Clavien-Dindo grade III complication
Hafez [19]	2005	Egypt	Posterior and anterior	35	Perineal approach via an inverted Y incision	2 perineal UTP, 2 endoscopic urethrotomy
Kardar [20]	1995	Saudi Arabia	Posterior	12	1 perineal approach, 11 APA, 4 cPBT, 7 pPBT	None reported
Orabi [24]	2008	Egypt	Posterior	50	47 PUA, 3 sUTP; 43 perineal approach; 4 abdominal approach a; 40 no PBT, 3 iPBT, 4 TPA	2 excision of urethral diverticulum
Podesta [27]	2015	Argentina	Posterior	49	Primary anastomosis: 28 perineal approach, 21 APA, 3 pPBT	4 AMR via PPPE, 1 AMR with urethral rerouting, 1 internal urethrotomy
Setato [29]	2021	Ethiopia	Posterior and anterior	19	19 pure perineal PUA without PBT	4 UTP, 3 UDL, 2 suprapubic cystostomy and waiting for redo surgery
Singh [30]	2014	India	Posterior	37	Urethral anastomosis: 29 pure preanal coronal approach, 8 preanal coronal + TPA	2 UTP, 3 UDL
Singla [31]	2008	India	Posterior	28	PUA: 27 perineal approach, 1 perineal + TPA	4 DVIU, 2 graft UTP, 1 redo anastomosis
Sreeranga [32]	2022	India	Posterior and anterior	181	UTP: 160 perineal approach, 17 abdominal approach + PBT, 4 combined approach	26 laser urethrotomy, 13 redo surgery=13
Voelzke [4]	2012	USA	Anterior and posterior	Anterior: 8 Posterior: 18	Anterior: 5 primary UTP, 3 U-BMG Posterior: PUA in all patients; 12 perineal only, 5 perineal + pPBT, 1 APA	3 DVIU
Wang [35]	2021	China	Posterior	8	3 perineal, 4 perineal + iPBT, 1 transperineal with CDS	None reported
Waterloos [5]	2018	Belgium	Posterior and anterior	13	12 PUA, 1graft UTP; 12 perineal approach only, 1 pPBT	1 redo via APA

AMR = anastamotic repair; APA = abdominoperineal approach; CDS = corporeal dissection and separation; CPD = complete pubic disjunction; DVIU = direct-vision internal urethrotomy; ; PBT = pubectomy; cPBT = complete PBT; iPBT = inferior PBT; pPBT = partial PBT; PPPE = perineal approach with partial pubectomy exposure; PUA = primary urethral anastamosis; TPA = transpubic approach; sUTP = substitution UTP; U-BMG = urethroplasty with buccal mucosal graft; UDL = urethral dilatation; UTP = urethroplasty.

a Approach not very clear for the sUTP cases.

**Table 2:** Overview of surgical details for late open reconstruction in girls

Study	Patients	Technique and approach
Ahmed [9]	5	Urethral reconstruction with flipped anterior bladder wall
Basiri [12]	3	Urethroplasty with abdominal approach and complete pubic disjunction
Sreeranga [32]	11	Meatoplasty (*n* = 2) Reconstruction via an abdominopelvic approach with partial pubectomy (*n* = 9)

The delay between trauma and definitive surgery was highly variable and was not mentioned in four studies [4,7,27,32]. In four studies the delay was at least 3 mo, but the maximum interval was not reported [5,16,29,35]. In just one study the delay was at least 6 mo [24]. The longest delay reported was 48 mo [8]. The initial management strategy showed great variability because some patients were treated in another center before referral. Nevertheless, initial treatment for most of the patients was placement of a suprapubic catheter.

A summary of the outcomes for early and late repairs is presented in Table 3. We analyzed data for 693 boys and 26 girls. Our aim was provide a comprehensive comparison by combining all cases reported to date; however, not all studies provided patient-level data.

**Table 3:** Overview of studies concerning early and or late open surgery

Study	Timing	Patients	Continence rate (%)	Stricture rate (%)	Redo surgery
Abdalla [7]^a^	Late	5	100	20	None reported
Aggarwal [8]	Late				
	Primary group	23	91	4	9% overall; 1 complex Monti reconstruction; 1 substitution urethroplasty, 1 UDL, 1 cystolithotomy
	Redo group	12	75	16	
Ahmed [9]	Late	5 (all girls)	100	0	20% overall; 1 augmentation cystoplasty + Mitrofanoff
Avanoglu [10]	Early	9	66	22	34% overall b; 4 reanastomosis, 2 balloon dilatation, 4 dilatation/endoscopic resection
	Late	4	50	50	
Balkan [11]	Early	12	100	17	6 dilatation/urethrotomy, 1 redo urethroplasty
	Late	8	88	38	6 dilatation/urethrotomy, 2 redo urethroplasty
Basiri [12]	Late	10 (7 boys, 3 girls)	80 (100% for boys)	0	None reported
Chaudhari [13]	Late	14	100	14	14% overall; 2 urethroplasty
Chukwubuike [14]	Late	11	100	9	9% overall; 1 urethroplasty
Das [15]	Late	10	100	30	20% overall; 3 UDL
El-Sheikh [16]	Late	15	NR	20	20% overall; 1 DVIU, 2 urethroplasty via APA
Garg [17]	Late	33	97	15	Not specifically mentioned, but 1 Clavien-Dindo grade III complication
Gündoğdu [18]	Early	12	75	58	None reported
Hafez [19]	Late	35	100	11	11% overall; 2 perineal urethroplasty, 2 endoscopic urethrotomy
Kardar [20]	Late	12	58	0	No redo reported
Nerli [21]	Early	5	100	40	2 IUT, 1 redo urethroplasty
	Late	10	100	30	3 IUT, 2 redo urethroplasty
Onen [23]	Early	8	88	25	40% overall^b^
	Late	16	81	25	
Orabi [24]	Late	50	100	10	12% overall; 2 excision of urethral diverticulum
Otgun [25]	Early	10	70	NR	Data not clear
	Late	1	100		
Podesta [26]	Early	1	75^b^	0^b^	25% overall^b^; 2 vaginoplasty
	Late	7			
Podesta [27]	Late	49	82	10	12% overall; 4 AMR via PPPE, 1 AMR with urethral rerouting, 1 IUT
Setato [29]	Late	19	NR	47	47% overall; 4 urethroplasty, 3 UDL, 2 SPC and waiting for redo surgery
Singh [30]	Late	37	100	13.5	6% overall; 2 urethroplasty, 3 UDL
Singla [31]	Late	28	100	25	25% overall; 4 DVIU, 2 graft urethroplasty, 1 redo anastomosis
Sreeranga [32]	Late	181 boys	92	22	22% overall; 26 laser urethrotomy, 13 redo surgery
		11 girls	91	0	None reported
Trachta [33]	Early	1	100	0	5 reanastomosis. 1 endoscopic transurethral resection
	Late	7	57	71	
Updahyaya [34]	Early	9	78	33	50% overall; 2 redo urethroplasty; 7 UDL; 1 awaiting redo
	Late	5	80	80	
Voelzke [4]	Late	26	NR	12	12% overall; 3 DVIU
Wang [35]	Late	8	100	0	None reported
Waterloos [5]	Late	13	92	8	1 redo via APA

AMR = anastamotic repair; APA = abdominoperineal approach; DVIU = direct-vision internal urethrotomy; IUT = internal urethrotomy; NR = not reported; PPPE = perineal approach with partial pubectomy exposure; SPC = suprapubic cystostomy; UDL = urethral dilatation.

a Data for the pediatric group of patients were extracted.

b No separate data.

All female cases underwent delayed repair, which precluded any comparison by timing. Among the male cases, 54 underwent early and 639 underwent delayed repair.

### 3.3 Endoscopic management

Among all the studies reviewed (regardless of whether they were categorized as deferred management, immediate management, or combined approaches), 14 reported on endoscopic management and outcomes for urethral trauma in children [4,5,10,13,16,17,19,21,[26], [27], [28],^32^,^34^,^35^]. Of these, nine mentioned endoscopic management as failed attempts before referral to the center for final repair [4,5,13,16,17,19,27,32,35].

One study by Podesta and Jordan [26] was on urethral injuries due to pelvic fracture in girls. Three patients were treated with endoscopic realignment and cystostomy, and seven underwent delayed open surgery. The authors reported urethral obliteration for all patients treated endoscopically. The same group reported on male urethral trauma in other studies and did not use endoscopic management in those cases [10,21,28,34]. Table 4 includes a summary of data from these four studies.

**Table 4:** Outcomes for cases with endoscopic management

Study	Trauma location	EM cases	Basis for EM selection	Treatment details	Outcomes
Avanoglu [10]	Posterior	14	SP	Endoscopic catheter realignment with no anastomosis	7 successful, 1 incontinent, 4 required TPU, 2 had no follow-up
Nerli [21]	Posterior	7	Associated injuries + SP	Endoscopic realignment (retrograde/antegrade)	All voiding well, no incontinence
Sanson [28]	Anterior	4/7	Unclear	4 immediate endoscopic realignment 7 endoscopic follow-up	7 successful
Upadhyaya [34]	Anterior	6	All patients with anterior trauma	UC with endoscopy (7 d for short, 6 wk for long injuries)	2 strictures (1 required multiple and 1 required few dilatations)

EM = endoscopic management; SP = surgeon’s preference; TPU = transpubic urethroplasty; UC = urethral catheterization.

### 3.4 Urethral stricture

Among the 31 articles reviewed, only one [22] did not investigate stricture incidence during follow-up. The proportion of patients who experienced stricture was 20% in the early repair group, and 19% in the delayed repair group. The difference was not statistically significant (*p* = 0.107).

The primary methods for assessing postoperative stricture are summarized in Table 5. Evaluation of urethral stricture relied predominantly on imaging and noninvasive functional assessments. The methods most frequently reported were voiding cystourethrography or retrograde urethrography (22 studies) [4,5,8,12,[14], [15], [16], [17],^19^,^20^,^23^,[25], [26], [27],^29^,[31], [32], [33], [34]] and uroflowmetry combined with measurement of postvoid residual volume (PVR; 14 studies) [4,5,8,10,13,19,[23], [24], [25],^28^,[31], [32], [33],^35^]. Four studies used more invasive procedures: urodynamic studies (UDS) [25,30] in two and routine endoscopy [15,25] in twos. Direct voiding observation was used in one study [29].

**Table 5:** Additional investigations used to evaluate strictures

Evaluation technique	Number of studies
VCU or retrograde urethrography	22 [3,4,7,11,[13], [14], [15], [16], [17], [18], [19],^22^,[24], [25], [26],^28^,[30], [31], [32], [33]]
Uroflowmetry with PVR measurement	14 [3,4,7,9,12,18,[22], [23], [24],^27^,[30], [31], [32],^34^]
Invasive urodynamics	2 [24,29]
Routine endoscopy	2 [14,24]
Voiding observation	1 [28]

PVR = postvoid residual volume; VCU = voiding cystourethrography.

**Fig. 2:**
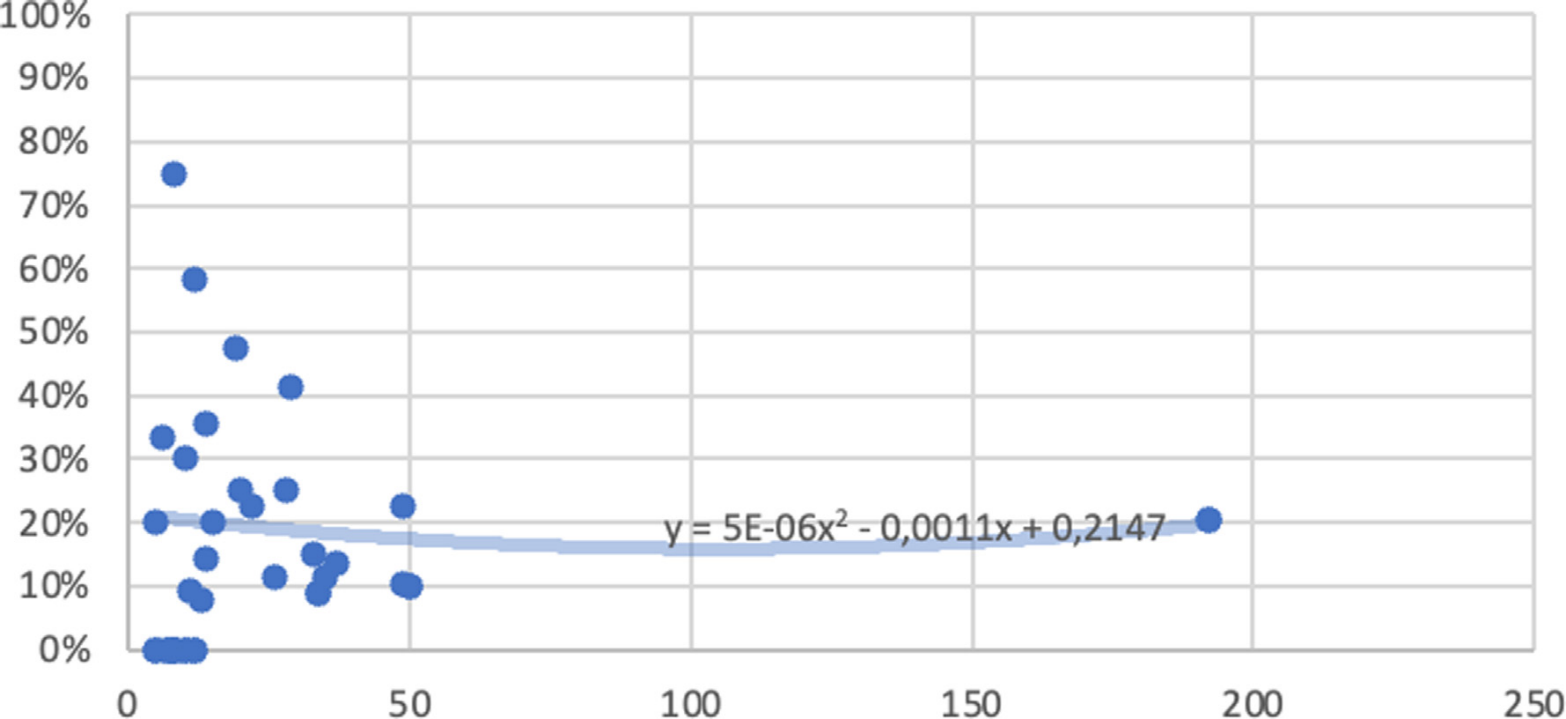
Scatterplot showing the proportion of patients with stricture as a function of cohort size in terms of the number of patients. Each dot represents a specific cohort. The trend curve, calculated using quadratic polynomial regression, depicts the overall relationship between the proportion of strictures and the cohort size.

The mean stricture rate across studies was 19% (range 0–80%). The variation in stricture rate did not appear to correlate with study size, as shown in Fig. 2, which provides the most visual representation of the results while remaining faithful to the reality of the highly variable findings across the studies.

### 3.5 Urinary continence

Of the 31 studies, 29 measured postprocedural urinary continence [5,[7], [8], [9], [10], [11],[13], [14], [15],[17], [18], [19], [20], [21], [22], [23], [24], [25], [26], [27], [28], [29], [30], [31], [32], [33], [34], [35]]. The proportion of patients who were continent after treatment was 83% in the early repair group, and 84% in the delayed repair group. The difference was not statistically significant (*p* = 0.052)

The definition of “dry” and “wet” before and after surgery was inconsistent and not in accordance with the International Children’s Continence Society (ICCS) guidelines [36]. Only Kardar et al [20] defined “dry” as daytime continence, and “wet” as total incontinence, enuresis nocturna, or stress incontinence.

The methods used to determine continence varied. Overall, the continence rate varied from 58% to 100%. Among girls, the continence rate varied from 75% to 100% [9,22,26]. Concerning boys, Aggarwal et al [8], reported a decrease in continence from 91% (21/23) after primary urethroplasty to 75% (9/12) after redo urethroplasty, perhaps related to combined pelvic fracture. Stress incontinence was the most common form in this study. Kardar et al [20] reported a continence rate of 58% (7/12) after combined perineal and abdominal urethroplasty.

### 3.6 Sexual function

Of the 31 studies, 17 measured erectile function postoperatively [5,7,8,10,[13], [14], [15],^20^,^21^,[25], [26], [27],[31], [32], [33], [34]]. The methods used varied significantly, with many studies relying on subjective reports from parents or children rather than standardized clinical assessments. Sreeranga et al [32] found that the incidence of erectile dysfunction (ED) was 29% (18/63) after primary repair, with a 30% improvement during follow-up, and 42% after redo surgery. Good erectile function was reported in 63% and 42% of cases primary and redo, respectively, according to direct questioning of parents and children. Using the validated Urethral Stricture Surgery Patient-reported Outcome Measure (USS-PROM), Waterloos et al [5] identified ED in 33% (2/6) of cases. Onen et al., found with Using an unvalidated tool, Onene et al [23] found that the ED incidence rate after urethral reconstruction was 15% for type B and 29% for type C pelvic fracture. Data from the three studies reporting detailed ED outcomes are summarized in Table 6.

**Table 6:** Erectile dysfunction after urethral trauma in children: assessment and outcomes

Study	Assessment technique used	ED outcomes
Sreeranga [32]	Ask the parents and child	**Primary repair** ED in 29% (30% of these improved during FU) Partial ED in 8% Good EF in 63% (16% of these developed partial ED during FU) **Redo PFUI repair** ED in 42% (19% of these improved during FU) Partial ED in 16% Good EF in 42%
Waterloos [5]	USS-PROM	2/6 boys evaluated reported ED
Onen [23]	Unvalidated questionnaire	ED after URC in 15% (4/27) of type B pelvic fracture cases and 29% (5/17) of type C pelvic fracture cases

ED = erectile dysfunction; EF = erectile function; FU = follow-up; PFUI = pelvic fracture urethral injury; URC = urethral reconstruction; USS-PROM = Urethral Stricture Surgery Patient-related Outcome Measure.

### 3.7 Quality of life

Only two studies mentioned systematic evaluation of the patient perspective using a survey that including questions on QoL [5,32]; one study used USS-PROMS [37], and one evaluated psychosexual outcomes [23]. However, only Onen et al [23] reported actual QoL outcomes: a psychiatric evaluation revealed psychiatric disorders in half of the patients and a lack of appropriate education during hospitalization. The number of urological procedures, the presence of long-term complications, and the number of hospitalizations were independent predictors for development of a psychological disease [23].

### 3.8 Follow-up

Follow-up ranged from 3 mo to 264 mo. The longest follow-up reported (up to 22 yr) was in the study by Podesta and Podesta [27]. Average, median, or mean follow-up duration could not be calculated as individual data were unavailable. Most studies mentioned follow-up extending to 5 yr after surgery, but unfortunately did not report outcomes at that time, raising questions about long-term results.

The nature of follow-up varied significantly across studies. Most described follow-up assessments that included a medical history, physical examination, and uroflowmetry ± PVR measurement in their methods section. However, only a few studies provided detailed uroflowmetry results, such as maximum flow velocity (Qmax) [7,13,19,21,25,28,33].

In some cases, video cystourethrography (VCUG) and/or a retrograde urethrogram (RUG) and/or cystoscopy were routine follow-up procedures, while in others these were performed only if stricture was suspected. UDS (or video UDS) was performed in a few studies, mainly in cases of urinary incontinence. Two studies performed UDS in all patients. Singh et al [30] found normal values in all except two cases: one had a high-pressure, low-capacity bladder, and the other had uninhibited detrusor contractions. Both patients were treated with anticholinergics. Otgun et al [25] compared 11 boys with posterior urethral trauma to 12 healthy control subjects and found significantly lower bladder capacity, compliance, and Qmax in the trauma group.

## 4 Discussion

Our aim was to provide insights into clinical treatment of urethral trauma in children, but data inconsistencies posed challenges. A classification scheme is also lacking. Classifications have been published by Goldman et al [38] and Moore et al [39], but these are not used in clinical practice. The guidelines for adults only use the anterior and posterior urethra for classification, as we did in the review. In this section we summarize key observations from the literature and guidance for future research.

### 4.1 Early versus late open reconstruction

No clear conclusions could be drawn regarding early versus late open reconstruction because of insufficient data, including the lack of a well-defined timeline for previous surgeries and inadequate reporting on techniques. Various surgical techniques were used among the studies, but often insufficient details were reported, and terms such as “operative urethral realignment” and “immediate operative urethral repair” were confusing. In studies discussing delayed open reconstruction [4,7,27,32], a definition of “delay” was frequently absent or inconsistent. If a late open reconstruction was chosen, placement of a suprapubic catheter at the time of the trauma was the usual approach. The delay until definitive surgery was highly variable (3–48 mo), with a delay of 3 mo often deemed acceptable without high-level evidence. Unfortunately, only one study provided an explanation [8], with the delay attributed to initial management performed elsewhere. Overall, early and late repairs appeared to result in similar rates of urinary continence and stricture formation, but conclusions are limited by incomplete data reporting.

### 4.2 Endoscopic management

The major issue for studies on endoscopic management is inconsistent outcome reporting and a lack of patient selection criteria. The data available suggest that endoscopic management might be suitable for some patients, but there is little information on patient selection, which was sometimes based on the surgeon’s preference [10,21]. Two studies favored endoscopic treatment for all anterior injuries [28,34], with one center avoiding this strategy for posterior cases [34]. Overall, endoscopic management was chosen by few centers and applied to a limited number of patients, with success mainly observed in male cases.

### 4.3 Urethral stricture

The methods for assessing and evaluating urethral stricture after surgery varied considerably across the studies. RUG or VCUG remained the preferred approach for visualizing the location and length of strictures, despite the radiation dose and invasiveness for children. However, these modalities are the most reliable for diagnosing recurrence. Invasive UDS appears to be underutilized, as only it was only performed in two studies [25,30]. UDS helps in distinguishing dysfunctional voiding from a true stricture, especially when using video UDS. Although this modality is invasive, it does not require general anesthesia (as cystoscopy does), but it might not be available in all clinics. By contrast, uroflowmetry with PVR measurement offers a simple, repeatable, and effective method for evaluation stricture occurrence in potty-trained children. The significant variation in stricture rates reported seemed to be independent of study size, suggesting that factors other than patient volume influenced these rates. Despite this variability, regression analysis for the overall trend revealed a consistent stricture rate of approximately 20% and a likelihood of convergence with increasing patient numbers.

### 4.4 Urinary continence

Regarding urinary continence, a major limitation is the lack of assessment and definition. Of the 31 studies included in the review, only one used a continence assessment tool [23], which highlights the need for more rigorous and uniform methods, such as validated questionnaires. The ICCS guidelines [36] delineate clear definitions for enuresis and incontinence, but these were rarely referenced, probably because many of the studies were published before the ICCS guidelines publication in 2016.

Urinary continence rates ranged from 58% to 100% following posterior urethral repair, and from 75% to 100% after for anterior urethral repair. These broad ranges suggest that trauma severity and individual patient characteristics play significant roles in outcomes. Numbers improved over the years, with a continence rate of 58% in 1995 [20] versus 92% in 2018 [5], suggesting positive evolution of surgical techniques.

Stress urinary incontinence was the most frequent type, but was often transient [22,27,30]. This can be explained by an improvement in the hypertrophic bladder muscle initially luxated by the urethral stricture and the proximity of the external sphincter mechanism to the surgical area.

Redo operations are associated with greater fibrosis and poor blood flow, which increase the risk of incontinence [8]. Despite these challenges, total incontinence was not observed after reconstruction.

### 4.5 Sexual function

ED following urethral trauma is underexplored because of the challenges in assessing sexual function in pediatric populations. Many affected boys are prepubescent, which limits the ability to assess erectile function. ED rates reported ranged from 0% to 46% [5,13,26,27,33]. The data may be biased, as boys with ED may be more likely to seek follow-up care, whereas those without ongoing problems are often lost to follow-up.

Hermosa et al [40] identified older age and urethral stricture length as potential risk factors for ED. Nontransecting urethroplasty techniques may reduce vascular damage and thus the risk of ED, but clinical evidence remains limited [41].

Waterloos et al [5] suggested that ED is more likely to attributable to the neurovascular damage caused by the trauma itself. Hermosa et al [40] also observed that ED following surgery is often transient, in agreement with findings reported by Sreeranga et al. [32].

### 4.6 Quality of life

The literature on pediatric urethral trauma lacks data on QoL. Only three studies mentioned QoL [5,23,32] and just two used a validated survey [5,32]. A major drawback is that the only validated survey is for individuals aged ≥16 yr. The USS-PROMS questionnaire, which was validated for adults in 2013 [37], does not address urinary incontinence or sexual function. These domains were added in the Dutch version by Verla et al [42]. There is no PROM for younger children.

Onen et al [23] highlighted disruption of education as another problem. Future studies should take this factor into consideration.

### 4.7 Follow-up

There is little consensus on follow-up duration or on what follow-up investigations are most relevant and necessary. Given that uroflowmetry with PVR measurement is a simple, noninvasive, and easily repeatable test, we believe that this should be a standard component of follow-up.

According to the European Association of Urology (EAU) guidelines on urethral strictures in adults, cystoscopy or RUG should be used to assess anatomic success after urethroplasty, as well as PROMs to assess subjective outcomes and validated questionnaires to evaluate sexual function [43]. As most recurrences occur within the first year, the EAU guidelines recommend at least 1 yr of follow-up, with tailoring of the protocol according to health costs and risks. No follow-up information is provided in other EAU guidelines (urological trauma, pediatric urology).

The American Urological Association (AUA) guidelines on urotrauma recommend monitoring for complications (stricture, ED, and urinary incontinence) for at least 1 yr, using uroflowmetry, RUG, cystoscopy, or a combination of these [44]. No specific strategy is recommended for tracking ED or incontinence. The AUA guidelines on urethral stricture advise monitoring for symptomatic recurrences, but no consensus has been reached on the optimal postoperative surveillance protocol.

### 4.8 Recommendations for further research

We propose several recommendations for future studies (Table 7), such as the development of PROMs for children with urethral trauma that include domains for sexual function and urinary continence. Parents should be counseled about the risk of ED and urinary incontinence, and encouraged to discuss these issues with their children at an age-appropriate time, seeking urological consultation if necessary. Greater awareness of the social impact of urethral trauma in children, including disruption of education, is needed.

**Table 7:** Suggestions for reporting urethral trauma in children

Topic	Recommendations
Study group	In studies with a combined patient group, consider presenting results separately by subgroups: • Females, male posterior urethra, male anterior urethra • Previous interventions: number and type of interventions • Patients before versus after puberty
Interventions	• Define interventions in detail • Consider giving details about drainage/urethral catheterization method before and after surgery • Define the timing of surgery, and the interval between trauma and surgery for delayed surgery • Define the selection criteria when using more than one technique
Outcome	• Present outcome data regarding stricture, urinary continence, sexual function, and quality of life • Define the evaluation methods for all outcomes o Surveys, clinical assessment strategies, radiological techniques, uroflowmetry, endoscopy o Consider using validated surveys or defining objective criteria (eg, stricture length) o Consider using patient-related outcome measures • Define the timeline for outcome assessment • Continue long term follow-up to evaluate sexual function during puberty and adulthood

## 5 Conclusions

Our review addressing a rare condition offers a structured overview of the literature on traumatic urethral trauma in children, covering 31 studies and 787 patients. Most striking is the poor quality and inconsistency of the evidence. Open surgery is more common than endoscopic management, and the overall postoperative stricture rate is approximately 20%. Urinary continence and sexual function are well preserved, as postoperative incontinence and ED are usually short-lived and recover in the majority of the patients over time. QoL is under-reported because of a lack of validated questionnaires.

We provide a dedicated table of recommendations to improve future studies, including the use of standardized definitions, validated pediatric PROMs, and consistent long-term follow-up.

  ***Author contributions:*** Rianne J.M. Lammers had full access to all the data in the study and takes responsibility for the integrity of the data and the accuracy of the data analysis.

  *Study concept and design*: Lammers, Tiryaki.

*Acquisition of data*: Lammers, Enache, Faraj, Waterschoot, Tiryaki.

*Analysis and interpretation of data*: Lammers, Enache, Faraj, Waterschoot, Tiryaki.

*Drafting of the manuscript*: Lammers.

*Critical revision of the manuscript for important intellectual content*: Lammers, Enache, Faraj, Waterschoot, Tiryaki.

*Statistical analysis*: Lammers, Faraj, Tiryaki.

*Obtaining funding*: None.

*Administrative, technical, or material support*: Lammers, Enache, Faraj, Waterschoot, Tiryaki.

*Supervision*: Lammers, Tiryaki.

*Other*: None.

  ***Financial disclosures:*** Rianne J.M. Lammers certifies that all conflicts of interest, including specific financial interests and relationships and affiliations relevant to the subject matter or materials discussed in the manuscript (eg, employment/affiliation, grants or funding, consultancies, honoraria, stock ownership or options, expert testimony, royalties, or patents filed, received, or pending), are the following: None.

  ***Funding/Support and role of the sponsor:*** None.

  ***Acknowledgments:*** We would like to thank Mirjam Harms and Vladimir Orlov for their help in the first stages of the literature search. We also thank Lisette ‘t Hoen for helping with the basic concept of the narrative review. We used AI (chat GPT) to improve the fluidity of certain parts of the manuscript.

## References

[1] Vetterlein M.W., Weisbach L., Riechardt S., Fisch M. (2019). Anterior urethral strictures in children: disease etiology and comparative effectiveness of endoscopic treatment vs. open surgical reconstruction. Front Pediatr.

[2] Radmayr C., Bogaert G., Bujons A. (2024).

[3] Wein A., Kavoussi L.R., Novick A.C., Partin A.W., Peters C.A. (2012). Campbell-Walsh urology.

[4] Voelzke B.B., Breyer B.N., McAninch J.W. (2012). Blunt pediatric anterior and posterior urethral trauma: 32-year experience and outcomes. J Pediatr Urol.

[5] Waterloos M., Verla W., Spinoit A.F. (2019). Urethroplasty for urethral injuries and trauma-related strictures in children and adolescents: a single-institution experience. J Pediatr Urol.

[6] Slim K., Nini E., Forestier D., Kwiatkowski F., Panis Y., Chipponi J. (2003). Methodological Index for Non-randomized Studies (MINORS): development and validation of a new instrument. ANZ J Surg.

[7] Abdalla M.A. (2008). A posterior sagittal pararectal approach for repair of posterior urethral distraction injuries. Eur Urol.

[8] Aggarwal S.K., Sinha S.K., Kumar A., Pant N., Borkar N.K., Dhua A. (2011). Traumatic strictures of the posterior urethra in boys with special reference to recurrent strictures. J Pediatr Urol.

[9] Ahmed S., Kardar A.H. (2000). Construction of a neourethra in girls: follow-up results. Pediatr Surg Int.

[10] Avanoglu A., Ulman I., Herek O., Ozok G., Gokdemir A. (1996). Posterior urethral injuries in children. Br J Urol.

[11] Balkan E., Kilic N., Dogruyol H. (2005). The effectiveness of early primary realignment in children with posterior urethral injury. Int J Urol.

[12] Basiri A., Shadpour P., Moradi M.R., Ahmadinia H., Madaen K. (2002). Symphysiotomy: a viable approach for delayed management of posterior urethral injuries in children. J Urol.

[13] Chaudhari R., Sharma A., Shaikh I., Andankar M., Pathak H. (2021). Safety and efficacy of trans-perineal urethroplasty for management of post-traumatic urethral strictures in pediatric age-group. Urol Int.

[14] Chukwubuike K.E., Enebe J.T., Nduagubam O.C. (2020). Urethral injury in children: experience in a teaching hospital in Enugu, Nigeria. Proc Singap Healthc.

[15] Das K., Charles A.R., Alladi A., Rao S., D’Cruz A.J. (2004). Traumatic posterior urethral disruptions in boys: experience with the perineal/perineal-transpubic approach in ten cases. Pediatr Surg Int.

[16] El-Sheikh M.G., Ziada A.M., Sadek S.Z., Shoukry I. (2008). Pediatric and adolescent transperineal anastomotic urethroplasty. J Pediatr Urol.

[17] Garg G., Kumar M., Singh M., Pandey S., Sharma A., Sankhwar S.N. (2019). Spectrum of management options for pediatric pelvic fracture urethral injury and outcome analysis: 12-year tertiary center experience. J Pediatr Urol.

[18] Gündoĝdu H., Tanyel F.C., Büyükpamukçu N., Hüyükpamukçu A. (1990). Primary realignment of posterior urethral ruptures in children. Br J Urol.

[19] Hafez A.T., El-Assmy A., Sarhan O., El-Hefnawy A.S., Ghoneim M.A. (2005). Perineal anastomotic urethroplasty for managing post-traumatic urethral strictures in children: the long-term outcome. BJU Int.

[20] Kardar A.H., Sundin T., Ahmed S. (1995). Delayed management of posterior urethral disruption in children. Br J Urol.

[21] Nerli R.B., Koura A.C., Ravish I.R., Amarkhed S.S., Prabha V., Alur S.B. (2008). Posterior urethral injury in male children: long-term follow up. J Pediatr Urol.

[22] Okur H., Kucikaydin M., Kazez A., Turan C., Bozkurt A. (1996). Genitourinary tract injuries in girls. Br J Urol.

[23] Onen A., Subasi M., Arslan H., Ozen S., Basuguy E. (2005). Long-term urologic, orthopedic, and psychological outcome of posterior urethral rupture in children. Urology.

[24] Orabi S., Badawy H., Saad A., Youssef M., Hanno A. (2008). Post-traumatic posterior urethral stricture in children: how to achieve a successful repair. J Pediatr Urol.

[25] Otgun I., Karnak I., Senocak M.E., Tanyel F.C., Ciftci A.O., Buyukpamukcu N. (2006). Late urodynamic findings after treating traumatic rupture of the posterior urethra in boys. BJU Int.

[26] Podesta M.L., Jordan G.H. (2001). Pelvic fracture urethral injuries in girls. J Urol.

[27] Podesta M., Podesta M. (2015). Delayed surgical repair of posttraumatic posterior urethral distraction defects in children and adolescents: long-term results. J Pediatr Urol.

[28] Sanson S., Ballouhey Q., Abbo O., Galinier P. (2013). Pediatric anterior urethral injuries: time to take stock. Prog Urol.

[29] Setato T., Mammo T.N., Wondemagegnehu B. (2021). Outcome of delayed perineal anastomotic urethroplasty in children with post-traumatic urethral stricture in a tertiary center, Addis Ababa, Ethiopia. Res Rep Urol.

[30] Singh A., Panda S.S., Bajpai M., Jana M., Baidya D.K. (2014). Our experience, technique and long-term outcomes in the management of posterior urethral strictures. J Pediatr Urol.

[31] Singla M., Jha M.S., Muruganandam K. (2008). Posttraumatic posterior urethral strictures in children—management and intermediate-term follow-up in tertiary care center. Urology.

[32] Sreeranga Y.L., Joshi P.M., Bandini M., Kulkarni S.B. (2022). Comprehensive analysis of paediatric pelvic fracture urethral injury: a reconstructive centre experience. BJU Int.

[33] Trachta J., Moravek J., Kriz J., Padr R., Skaba R. (2016). Pediatric bulbar and posterior urethral injuries: operative outcomes and long-term follow-up. Eur J Pediatr Surg.

[34] Upadhyaya M., Freeman N.V. (2002). Management of traumatic urethral disruption in children: Oman experience, 1988-2000. J Pediatr Surg.

[35] Wang L., Chen J., Lv R. (2022). Pelvic fracture urethral distraction defects in preschool boys: how to recognize and manage?. Urology.

[36] Austin P.F., Bauer S.B., Bower W. (2016). The standardization of terminology of lower urinary tract function in children and adolescents: update report from the standardization committee of the International Children’s Continence Society. Neurourol Urodyn.

[37] Jackson M.J., Chaudhury I., Mangera A. (2013). A prospective patient-centred evaluation of urethroplasty for anterior urethral stricture using a validated patient-reported outcome measure. Eur Urol.

[38] Goldman S.M., Sandler C.M., Corriere J.N., McGuire E.J. (1997). Blunt urethral trauma: a unified, anatomical mechanical classification. J Urol.

[39] Moore E.E., Cogbill T.H., Jurkovich G.J. (1992). Organ injury scaling. III: chest wall, abdominal vascular, ureter, bladder, and urethra. J Trauma.

[40] Hermosa P.C., Campos-Juanatey F., Malo R.V., Gómez M.A.C., Baños J.L.G., Trauma and Reconstructive Urology Working Party of the European Association of Urology Young Academic Urologists (2021). Sexual function after anterior urethroplasty: a systematic review. Transl Androl Urol.

[41] Verla W., Waterloos M., Waterschoot M., Van Parys B., Spinoit A.F., Lumen N. (2020). VeSpAR trial: a randomized controlled trial comparing vessel-sparing anastomotic repair and transecting anastomotic repair in isolated short bulbar urethral strictures. Trials.

[42] Verla W., Waterloos M., Lumen N. (2017). Urethroplasty and quality of life: psychometric validation of a Dutch version of the Urethral Stricture Surgery Patient Reported Outcome Measures. Urol Int.

[43] Lumen N., Campos-Juanatey F., Dimitropoulos K. (2024).

[44] Morey A.F., Brandes S., Dugi D.D. (2014). Urotrauma: AUA guideline. J Urol.

