# Supplementary material for: Noniatrogenic Urethral Trauma in Children: A Narrative Review
Source: Eur Urol Open Sci. 2025 Oct 13;82:41–51. doi: 10.1016/j.euros.2025.09.016 (PMC12549530; doi:10.1016/j.euros.2025.09.016)
Supplement: Supplementary Data 1 [file mmc1.docx]

**Supplementary material: MINORS score per study**

| **First author** | **Year of publication** | **MINORS Score** |
| --- | --- | --- |
| Abdalla | 2008 | 8/16 |
| Aggarwal | 2011 | 6/16 |
| Ahmed | 2000 | 6/16 |
| Avanoglu | 1996 | 4/16 |
| Balkan | 2005 | 13/24 |
| Basiri | 2002 | 8/16 |
| Chaudhari | 2021 | 12/16 |
| Chuwubuike | 2020 | 9/16 |
| Das | 2004 | 9/16 |
| El-Sheikh | 2008 | 9/16 |
| Garg | 2019 | 10/16 |
| Gundogdu | 1990 | 6/16 |
| Hafez | 2005 | 8/16 |
| Kardar | 1995 | 8/16 |
| Nerli | 2008 | 5/16 |
| Okur | 1996 | 7/16 |
| Onen | 2005 | 10/16 |
| Orabi | 2008 | 8/16 |
| Otgun | 2005 | 15/24 |
| Podesta | 2001 | 8/16 |
| Podesta | 2015 | 8/16 |
| Sanson | 2013 | 5/16 |
| Setato | 2021 | 8/16 |
| Singh | 2014 | 7/16 |
| Singla | 2008 | 7/16 |
| Sreeranga | 2022 | 7/16 |
| Trachta | 2016 | 11/16 |
| Upadhyaya | 2002 | 5/16 |
| Voelzke | 2012 | 7/16 |
| Wang | 2021 | 8/16 |
| Waterloos | 2019 | 11/16 |
